# *MdVQ37* overexpression reduces basal thermotolerance in transgenic apple by affecting transcription factor activity and salicylic acid homeostasis

**DOI:** 10.1038/s41438-021-00655-3

**Published:** 2021-10-01

**Authors:** Qinglong Dong, Dingyue Duan, Wenqian Zheng, Dong Huang, Qian Wang, Xiaoran Li, Ke Mao, Fengwang Ma

**Affiliations:** State Key Laboratory of Crop Stress Biology for Arid Areas/Shaanxi Key Laboratory of Apple, College of Horticulture, Northwest A & F University, 712100 Yangling, Shaanxi China

**Keywords:** Heat, Plant physiology, Plant hormones

## Abstract

High temperature (HT) is one of the most important environmental stress factors and seriously threatens plant growth, development, and production. VQ motif-containing proteins are transcriptional regulators that have been reported to regulate plant growth and developmental processes, including responses to biotic and abiotic stresses. However, the relationships between VQ motif-containing proteins and HT stress have not been studied in depth in plants. In this study, transgenic apple (*Malus domestica*) plants overexpressing the apple VQ motif-containing protein-coding gene (*MdVQ37*) were exposed to HT stress, and the transgenic lines exhibited a heat-sensitive phenotype. In addition, physiological and biochemical studies revealed that, compared with WT plants, transgenic lines had lower enzymatic activity and photosynthetic capacity and lower amounts of nonenzymatic antioxidant system metabolites under HT stress. Transcriptome analysis revealed 1379 genes whose expression differed between the transgenic lines and WT plants. GO and KEGG pathway analyses showed that transcription factor activity and plant hormone signaling pathways were differentially influenced and enriched in the transgenic lines. Salicylic acid (SA) content analysis indicated that overexpression of *MdVQ37* reduced the content of endogenous SA by regulating the expression of SA catabolism-related genes, which ultimately resulted in disruption of the SA-dependent signaling pathway under HT stress. The application of SA slightly increased the survival rate of the transgenic lines under HT stress. Taken together, our results indicate that apple MdVQ37 has a regulatory function in basal thermotolerance by modulating the activity of transcription factors and SA homeostasis. Overall, this study provides novel insights that improve our understanding of the various functions of VQ motif-containing proteins.

## Introduction

As sessile organisms, plants cannot escape the effects of environmental stress during their growth and development, and high temperature (HT) is among the major types of environmental stress. As global warming intensifies, the frequency and extent of extreme HTs continue to increase, posing a major threat to plant growth and development, crop yields, and food security^1,2^. Plants exhibit a heat shock response under HT stress. They also have the ability to survive direct exposure to extreme HT (i.e., basal thermotolerance) and can also increase their tolerance to lethal HT stress after acclimation to nonlethal HT stress for a certain period of time (i.e., acquired thermotolerance)^3–5^.

After plants are subjected to HT stress, a series of damaging events occur. First, HT stress causes severe damage to thylakoids, severely limiting the photosynthetic electron transfer rate of plant photosystems (PSs) and the activity of several related enzymes and rapidly inhibiting photosynthesis in plants^6^. Second, HT stress disrupts the oxygen-evolving complex, resulting in an imbalance during the energy conversion between the photosystem II (PSII) oxygen-evolving complex and the receptor site of the photosystem, resulting in irreversible damage to PSII and greatly reducing its activity^6,7^. HT can also cause plant stomata to close and affects leaf stomatal conductance (gs) and intercellular CO_2_ concentrations^8,9^. Furthermore, HT stress can disrupt the dynamic balance between harmful reactive oxygen species (ROS) production and removal in plants, and the accumulation of superoxide anions (O_2_.−), hydrogen peroxide (H_2_O_2_), singlet oxygen species (^1^O_2_), and hydroxyl radicals (OH–) increases the degree of membrane lipid peroxidation, ultimately leading to oxidative stress^6,10^.

Plants have evolved complex and diverse strategies to accommodate HT stress. Initially, plants evolved antioxidant defense systems under HT stress to reduce the harmful effects of oxidative stress^6^. Plant antioxidant defense systems include nonenzymatic and enzymatic ROS-scavenging systems. The nonenzymatic ROS-scavenging system consists mainly of carotenoids, ascorbic acid (AsA), and glutathione (GSH), whereas the enzymatic system mainly includes peroxidase (POD), catalase (CAT), superoxide dismutase (SOD), glutathione reductase (GR), ascorbate peroxidase (APX), dehydroascorbate reductase (DHAR) and monodehydroascorbate reductase (MDHAR)^6,11^. Furthermore, under HT stress, plants can accumulate osmotic adjustment substances to maintain plant cell osmotic pressure and reduce stress-induced damage. Osmotic adjustment substances include proline, soluble sugars, soluble protein, betaine, and polyamines^6^. In addition, when plants are subjected to HT stress, their response at the molecular level is to synthesize a variety of heat-shock proteins and heat stress-responsive transcription factors (TFs), thereby enhancing the ability of plants to resist HT stress^10^. Heat stress-responsive TFs include HSF, AP2/ERF, bZIP, MYB/MYC, NAC (NAM, ATAF, and CUC), and WRKY TFs^12–16^.

Several studies have indicated that salicylic acid (SA) is an important phenolic compound that acts as an endogenous plant signaling molecule and is involved in the regulation of plant growth, development and defense responses^17^. The function of SA in plant-pathogen interactions has been well documented. In addition to biotic stress, SA participates in the activation of several plants abiotic responses^17–20^. For example, SA can help counter the adverse effects of heat and salt stress on photosynthesis, AsA-GSH, and the activities of ROS-scavenging enzymes in wheat and choy sum, respectively^18,20^. Overexpression of *LcSABP2*, which is the SA-binding protein 2-encoding gene from *Lycium chinense*, in transgenic tobacco lines resulted in lower ROS levels, higher endogenous SA contents, and photosynthetic capacities, and improved tolerance to poor nutrient stress^21^.

In 2002, the first VQ motif-containing protein (SIGMA FACTOR-BINDING PROTEIN 1, VQ23/SIB1) was discovered in *Arabidopsis*^22^. A series of subsequent studies revealed that VQ motif-containing proteins participate not only in the regulation of plant growth but also in developmental processes^23^. For example, the *vq14/iku1* mutant exhibits a small-seed phenotype, and VQ18 and VQ26 were responsive to abscisic acid (ABA) treatment, indicating that these VQ motif-containing proteins regulate seed size and germination^24,25^. The *vq29* loss-of-function mutant displays decreased hypocotyl elongation under white light and low-intensity far-red light^26^. These results suggest that VQ29 negatively regulates seedling photomorphogenesis^26^. The triple-mutant *wrky2-1 wrky34-1 vq20-1* presents compromised pollen germination, development, and tube growth, suggesting that VQ20 affects plant male gametogenesis^27^. In addition, VQ motif-containing proteins have been found to participate in the plant defense response and stress tolerance. For instance, *amiR-vq12 vq29* double-mutants present significantly enhanced disease resistance to the fungus *Botrytis cinerea*, whereas overexpression of *VQ12* or *VQ29* increased susceptibility to *B. cinerea*^28^. These results suggested that VQ12 and VQ29 play negative regulatory roles in the response to *B. cinerea* infection by functioning as negative regulators of plant basal defense against *B. cinerea*. Compared with wild-type (WT) plants, mutant *vq9* plants show increased tolerance to salt stress, whereas the overexpression of *VQ9* renders plants hypersensitive to salt stress^29^. These results indicated that VQ9 functions as a negative regulator of the response to salt stress. To date, few studies have functionally analyzed the biological functions of VQ motif-containing proteins in the HT stress response^30^.

Apple (*Malus domestica*) trees are one of the most important fruit tree species in temperate regions of the world and tolerate a wide range of cultivation areas. However, HT stress severely reduces apple growth, development, quality, and production. Here, we examined the expression patterns of various apple *MdVQ* genes and found that *MdVQ37* was clearly downregulated in response to HT stress. In addition, *MdVQ37*-overexpressing apple plants were used to elucidate the biological function of this gene in the HT stress response. We found that *MdVQ37* overexpression reduced basal thermotolerance in transgenic apple lines. This negative response was related to a production of greater amounts of toxic ROS, a reduction in the AsA-GSH level, and interference with photosynthesis compared with those in WT plants. Additional analysis suggested that this negative effect stemmed from changes in TF activity and SA accumulation levels. Generally, these findings provide novel insights that improve our understanding of the multiple functions of VQ motif-containing proteins. At the same time, these findings may have important applications in the breeding of horticultural plants in the face of the frequent occurrence of extreme climate conditions.

## Results

### Analysis of the expression of *MdVQ* genes under HT stress

In a previous study, we found and identified VQ family members in the apple genome and thoroughly analyzed the evolution and structure of apple *MdVQ* genes, as well as the biological functions of some MdVQ members^31^. In this study, we analyzed the expression levels of various *MdVQ* genes under HT stress via RT-qPCR. The expression levels of *MdVQ1*, *MdVQ2*, *MdVQ12,* and *MdVQ25* were significantly upregulated in response to HT stress, whereas those of *MdVQ4*, *MdVQ6*, *MdVQ16*, *MdVQ20*, *MdVQ26*, *MdVQ31*, *MdVQ34*, *MdVQ37*, *MdVQ38*, *MdVQ42,* and *MdVQ44* were significantly downregulated (Fig. S1). Among these genes, the expression of *MdVQ12* increased 8.45-fold after treatment for 4 h compared with that of the control (Fig. S1); the expression level of *MdVQ37* decreased 49-fold after treatment for 8 h compared with that of the control (Fig. S1). Taken together, these results suggest that multiple VQ genes are involved in the response to HT stress in apples.

### Overexpression of *MdVQ37* increased sensitivity to HT stress in apple

**Fig. 1:**
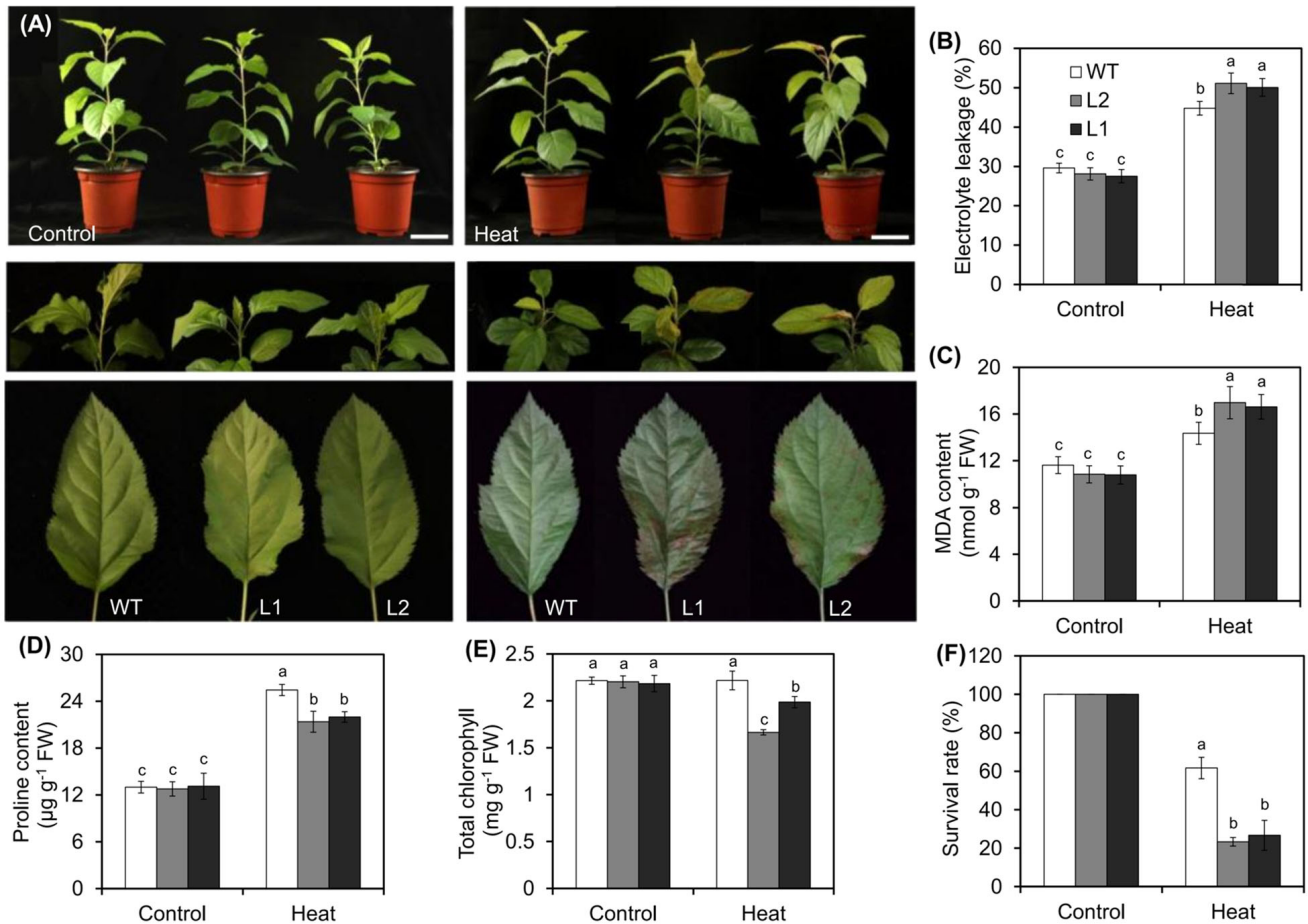
Overexpression of *MdVQ37* increases sensitivity to HT stress in transgenic apple lines. **A** Phenotypes of WT plants and *MdVQ37* transgenic lines under HT stress. Ninety-day-old WT plants and transgenic lines of uniform size were exposed to HT stress for 6 h in red plastic pots (10 × 10 × 8.5 cm). The plants were imaged after 0 and 6 h of HT stress. **B** Electrolyte leakage (EL), (**C**) malondialdehyde (MDA) content, (**D**) proline content, (**E**) chlorophyll concentration, and (**F**) survival rate of WT plants and *MdVQ37* transgenic lines subjected to HT stress or not. The data are presented as the means ± SEs of three biological experiments. The different letters represent significant differences among the treatments on each date (one-way analysis of variance (ANOVA) followed by Duncan’s test, *p* < 0.05)

Based on the expression patterns of *MdVQ* genes under HT stress (Fig. S1) and the screening results of interactions between MdVQ motif-containing proteins and MdWRKY transcription factors^31^, three *MdVQ* genes, *MdVQ6*, *MdVQ34,* and *MdVQ37*, were selected for subsequent genetic transformation and functional identification in apple. Due to the extremely low efficiency of transgene expression in apple plants, only two *MdVQ37*-overexpressing lines (L1 and L2) were obtained, and no lines with significantly reduced *MdVQ37* expression were obtained via genetic transformation. Compared with those in WT plants, the transcript levels of *MdVQ37* in L1 and L2 constitutively increased by 124.88-fold and 85.36-fold, respectively (Fig. S2). After exposure to 48 °C for 6 h, the top leaves of the transgenic lines wilted severely and displayed necrotic spots. In contrast, the top leaves of WT plants showed symptoms of only slight dehydration, and the fully expanded leaves of the lower parts of the plants remained green and vigorous (Fig. 1A). Electrolyte leakage (EL) and malondialdehyde (MDA) levels were used as indicators of abiotic stress-induced membrane damage^9,11^. Thus, we measured the levels of EL in the WT plants and transgenic lines under HT stress. The levels of EL increased significantly due to the effect of HT stress, and the EL levels in the WT plants were lower than those in the transgenic lines (Fig. 1B). Similar trends in the MDA content were observed in the WT plants and transgenic lines under HT stress (Fig. 1C), which indicated that the transgenic lines sustained substantially more damage from HT stress. In addition, although HT stress caused an increase in proline content in the leaves, we found that the proline content in the transgenic lines was significantly lower than that in the WT plants (Fig. 1D). Following exposure to HT, the total chlorophyll concentration of the WT plants did not change, but the total chlorophyll concentration of the transgenic lines significantly decreased (Fig. 1E). To further evaluate the HT stress tolerance of the *MdVQ37* transgenic lines, we subjected the WT plants and transgenic lines to 48 °C for 24 h (Fig. S3). After the plants were allowed to recover at 24 °C for 48 h, we determined the survival rate of the WT plants and transgenic lines. The results in Fig. 1F reveal that the survival rate of the transgenic lines and WT plants was significantly reduced, but the survival rate of the WT plants was much higher than that of the transgenic lines. All of these findings indicated that the transgenic lines sustained increased physiological damage under HT stress, implying that *MdVQ37* plays a negative role in the response to HT stress in apples.

### Overexpression of *MdVQ37* increased ROS accumulation and reduced antioxidant enzyme activities under heat stress

**Fig. 2:**
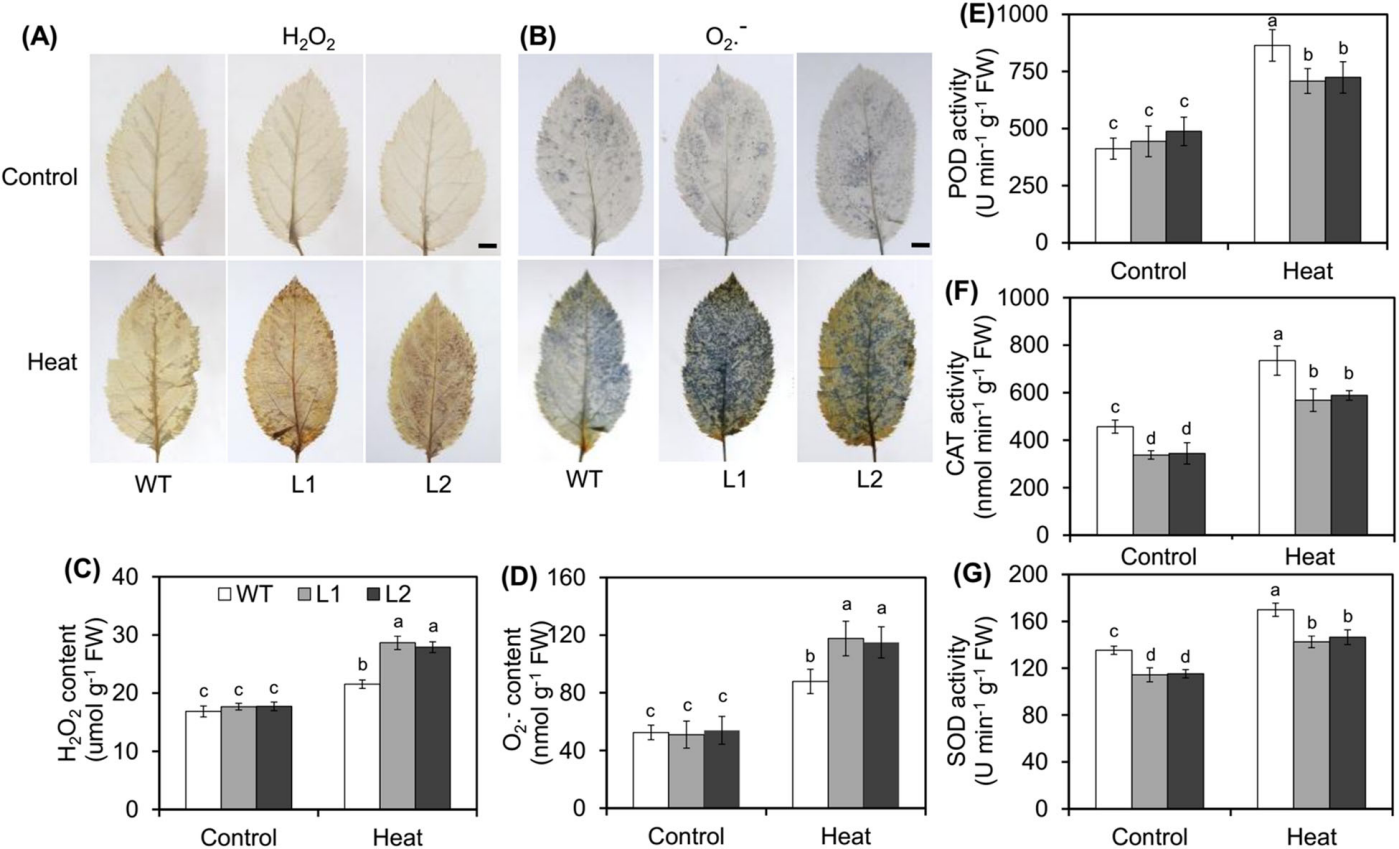
Changes in ROS accumulation and antioxidant enzyme activity in WT plants and *MdVQ37* transgenic lines under HT stress. Accumulation of (**A**) H_2_O_2_ and (**B**) O_2_·− in the *MdVQ37* transgenic lines and WT plants as revealed by histochemical staining with (**A**) DAB and (**B**) NBT. **C** H_2_O_2_ content, (**D**) O_2_·− content, (**E**) POD activity, (**F**) CAT activity, and (**G**) SOD activity in WT plants and *MdVQ37* transgenic lines subjected to HT stress or not. The data are presented as the means ± SEs of three biological experiments. The different letters represent significant differences among the treatments on each date (one-way analysis of variance (ANOVA) followed by Duncan’s test, *p* < 0.05)

The excessive accumulation of ROS such as O_2_·− and H_2_O_2_ can lead to oxidative stress, damage macromolecules, and cell membrane lipid structures, and cause harmful effects in plants^32^. For this reason, we investigated the effects of the overexpression of *MdVQ37* by measuring the in situ accumulation of O_2_·− and H_2_O_2_ via nitro blue tetrazolium (NBT) and 3,3’-diaminobenzidine (DAB) histochemical staining, respectively. The leaves of the transgenic lines showed stronger brown or blue spots after HT stress than did the leaves of the WT plants (Fig. 2A, B). This result implies that the transgenic lines accumulated more ROS than the WT plants did under HT stress. The quantitative measurements of O_2_·− and H_2_O_2_ were consistent with the above data (Fig. 2C, D). SOD, CAT, and POD are three effective antioxidant enzymes involved in ROS scavenging^33^. Therefore, the activities of SOD, CAT, and POD enzymes were measured in the transgenic lines and WT plants. The activities of the CAT and SOD enzymes in WT plants were significantly higher than those in transgenic lines under normal conditions (Fig. 2E–G). After 6 h of heat exposure, the activities of these three enzymes increased significantly in the apple plants. However, this increase was significantly lower in the transgenic lines compared with the WT plants (Fig. 2E–G). Overall, our data indicated that overexpression of *MdVQ37* reduced antioxidant enzyme activities, thereby resulting in excessive ROS production under HT stress.

### Overexpression of *MdVQ37* reduced the efficiency of the AsA-GSH cycle under HT stress

**Fig. 3:**
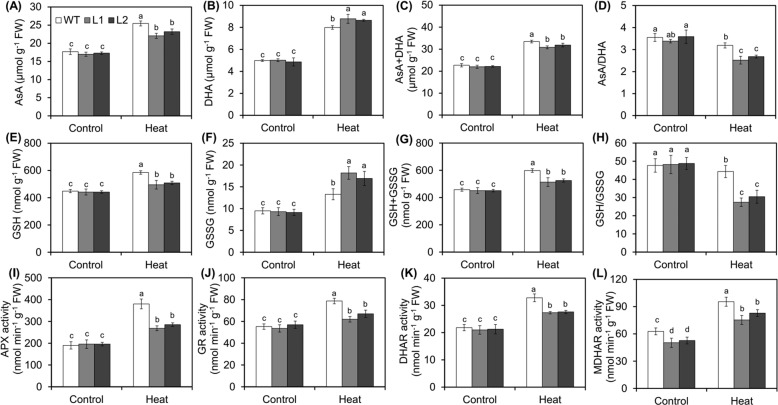
Changes in antioxidant contents and activities of the main antioxidative enzymes in the ascorbate-glutathione (AsA-GSH) cycle under HT stress. **A** AsA content, (**B**) DHA content, (**C**) AsA + DHA content, (**D**) AsA/DHA content, (**E**) GSH content, (**F**) GSSG content, (**G**) GSH + GSSG content, (**H**) GSH/GSSG, (**I**) APX activity, (**J**) GR activity, (**K**) DHAR activity, and (**L**) MDHAR activity in WT plants and *MdVQ37* transgenic lines subjected to HT stress or not. The data are presented as the means ± SEs of three biological experiments. The different letters represent significant differences among the treatments on each date (one-way analysis of variance (ANOVA) followed by Duncan’s test, *p* < 0.05)

It is known that the AsA-GSH cycle is an important antioxidant defense system that works against oxidative damage in plants^9,34^. Therefore, to further study the effect of MdVQ37 on the AsA-GSH cycle under HT stress, we determined the concentrations of AsA and GSH in the transgenic lines and WT plants. Under normal conditions, the total ascorbate (AsA + dehydroascorbate (DHA)) content, total glutathione (GSH + glutathione disulfide (GSSG)) content, and the ratios of AsA/DHA and GSH/GSSG were at the same levels in the transgenic lines and WT plants (Fig. 3A–H). However, after 6 h of exposure to HT stress, the contents of AsA, DHA, and AsA + DHA in the WT plants and transgenic lines significantly increased, although the contents of AsA and AsA + DHA in the transgenic lines were significantly lower than those in the WT plants (Fig. 3A, C). In addition, the DHA content in the transgenic lines was significantly higher than that in the WT plants (Fig. 3B), and the AsA/DHA ratio in the transgenic lines decreased more markedly than it did in the WT plants under HT stress (Fig. 3D). The levels of GSH, GSSG, and GSH + GSSG and the GSH/GSSG ratio exhibited similar trends (Fig. 3E–H). Moreover, similar to the changes in the contents and trends of AsA and GSH, the measurements of the activities of the APX, GR, DHAR, and MDHAR enzymes involved in the AsA-GSH cycle in the transgenic lines and WT plants revealed that after 6 h of exposure to HT stress, the activities were significantly reduced in the transgenic lines compared with the WT plants (Fig. 3I–L). Taken together, these results revealed that the overexpression of *MdVQ37* in transgenic apple plants attenuated the antioxidant capacity of AsA-GSH recycling under HT stress.

### Overexpression of *MdVQ37* aggravated the photoinhibition of the photosynthetic system under HT stress

**Fig. 4:**
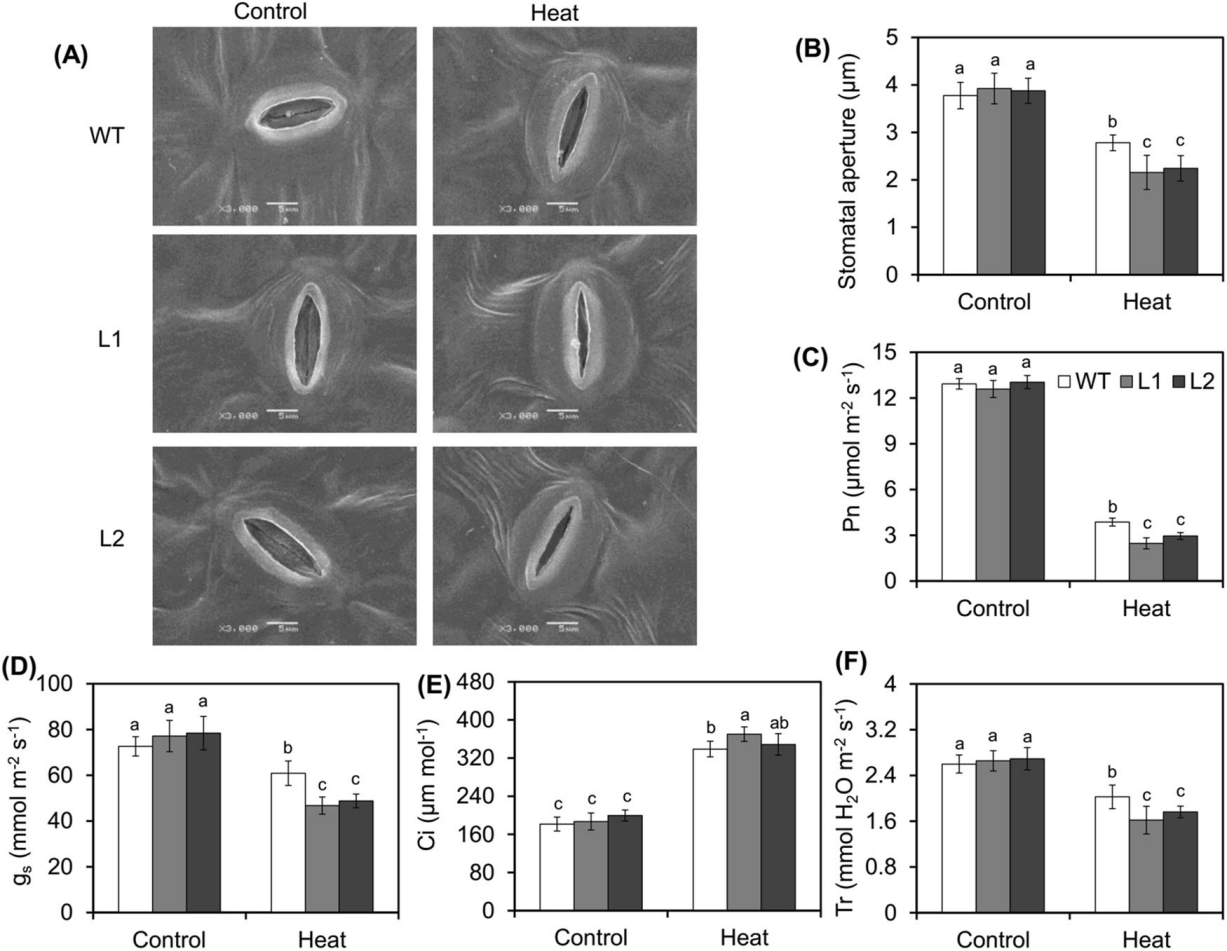
Stomatal behavior and photosynthetic capacity of WT plants and *MdVQ37* transgenic lines under HT stress. **A** SEM images of stomata from WT plants and *MdVQ37* transgenic lines after 4 h of HT treatment. (**B**) Changes in stomatal apertures in **A**. The (**C**) net photosynthesis (Pn), (**D**) stomatal conductance (g_s_), (**E**) intercellular CO2 concentration (Ci), and (**F**) transpiration rate (Tr) of WT plants and *MdVQ37* transgenic lines were measured under normal temperature or after 24 h of recovery after HT treatment. The data represent the means ± SEs of eight replications. The different letters represent significant differences among the treatments on each date (one-way analysis of variance (ANOVA) followed by Duncan’s test, *p* < 0.05)

HT stress causes leaf stomata to close, thereby reducing photosynthetic efficiency and affecting plant growth. Therefore, we compared the stomatal morphology between WT plants and transgenic lines under HT stress. No difference in the stomatal opening was observed between the WT plants and transgenic lines under normal-temperature conditions (Fig. 4A, B). However, after 4 h of heat exposure, the stomatal shrinkage of the transgenic lines increased compared with that of the WT plants (Fig. 4A, B). Furthermore, a comparison of the measurements of photosynthesis-related parameters between the transgenic lines and WT plants under HT stress revealed, that under normal conditions, there were no differences in net photosynthesis (Pn), stomatal conductance (g_s_), intercellular CO_2_ concentration (Ci), or transpiration rate (Tr) between the WT plants and transgenic lines (Fig. 4C–F). After 6 h of exposure to HT stress followed by recovery for 24 h, the Pn value decreased sharply. The Pn value of the WT plants was approximately 29.9% that of the controls, whereas that of the transgenic lines was approximately 19.5 and 22.6% that of those of the controls (Fig. 4C). Consistent with the Pn data, the g_s_ and Tr data showed similar trends after the recovery period (Fig. 4D, F). However, the change in the Ci value between the WT plants and transgenic lines during the recovery period was small (Fig. 4E). Taken together, the above results indicate that the photosynthetic function of *MdVQ37* was more compromised in the transgenic lines than in the WT plants under HT stress.

**Fig. 5:**
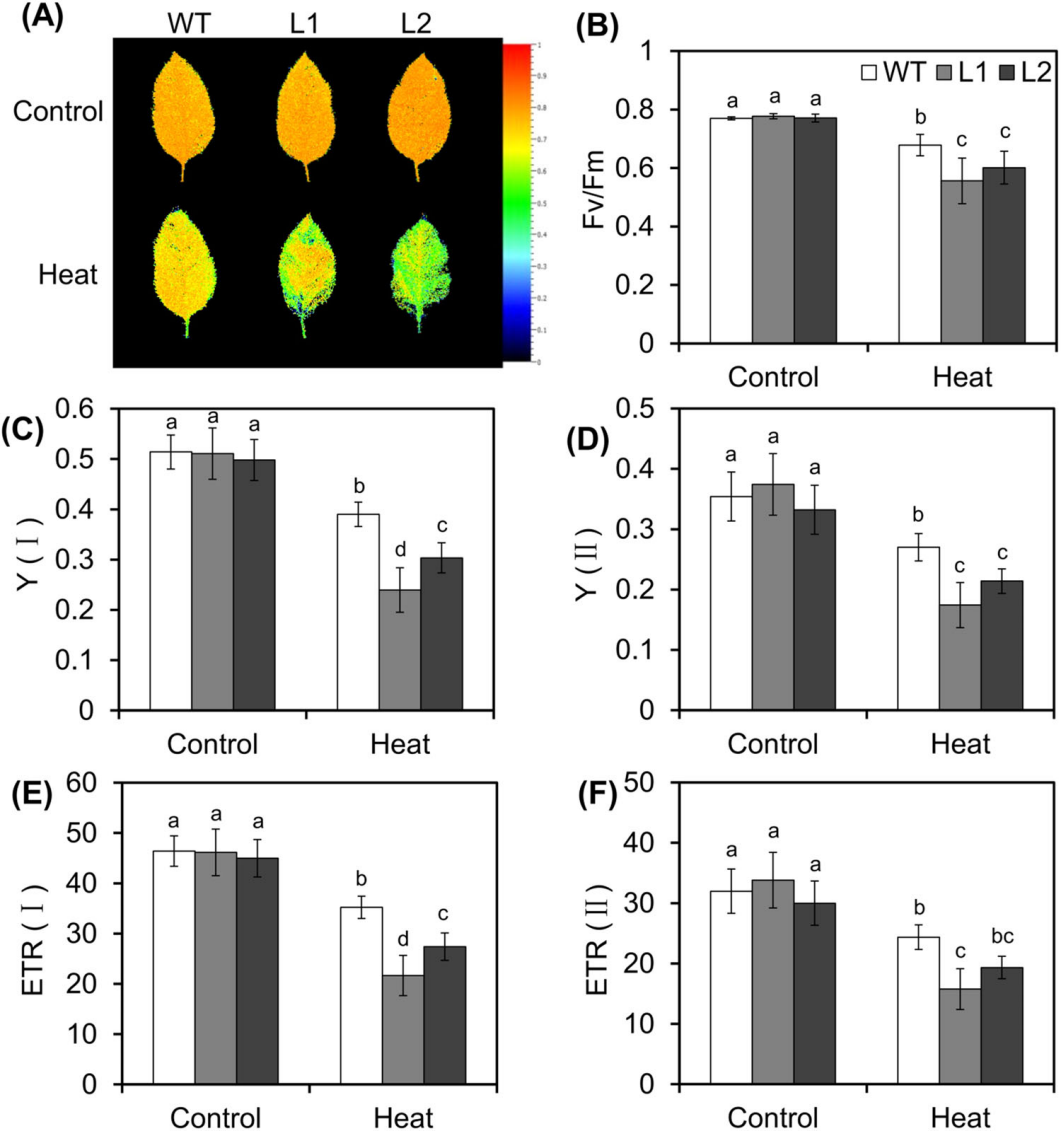
Capacity of PSI and PSII in WT plants and *MdVQ37* transgenic lines after HT treatment. **A** Chlorophyll fluorescence images (Fv/Fm) and (**B**) Fv/Fm ratios of *MdVQ37* transgenic lines and WT plants under normal temperature or after 24 h of recovery after HT treatment. The effective quantum yield and electron transport rate of PSI [Y(I) (**C**) and ETR (I) (**D**)] and PSII [Y(II) (**E**) and ETR (II) (**F**)] were measured at a normal temperature or after 12 h of recovery after high-temperature treatment. The data represent the means ± SEs of eight replications. The different letters represent significant differences among the treatments on each date (one-way analysis of variance (ANOVA) followed by Duncan’s test, *p* < 0.05)

Analysis of the maximum photochemical efficiency of photosystem II (PSII) photochemistry (variable fluorescence/maximum fluorescence (Fv/Fm)), a valuable criterion for studying the effect of abiotic stress on plant photosynthesis^35,36^, via fluorescence imaging showed that transgenic lines and WT plants initially exhibited similar fluorescence intensities under normal conditions. However, the fluorescence of the transgenic lines became more intense than that of the WT plants after 24 h of recovery from HT stress (Fig. 5A). Consistent with the fluorescence imaging results, the Fv/Fm ratio, a parameter representing the health and growth of plants, of the transgenic lines was equivalent to that of the WT plants under control conditions but was significantly lower after 24 h of recovery from HT stress (Fig. 5B). Further analysis of the photochemical changes in PSI and PSII using a Dual-PAM-100 measurement system to evaluate the energy changes in PSI and PSII^37^ revealed that, initially, the transgenic and WT plants exhibited similar Y(I), ETR(I), Y(II), and ETR(II) values under normal conditions. However, these indicators significantly decreased after 24 h of recovery from HT stress (Fig. 5C–F). Specifically, the Y(II) values of line 1 and line 2 were 0.64 and 0.79 times that of WT plants under HT stress, respectively (Fig. 5D). Similar trends were also found in the changes in ETR (I), ETR (II), and Y (I) under HT stress (Fig. 5C, E, F). Overall, these results revealed that overexpression of *MdVQ37* in the transgenic plants had negative effects on photochemical reactions, including those of both PSI and PSII, under HT-stress conditions.

### *MdVQ37* overexpression affects TF activity and plant hormone signaling

**Fig. 6:**
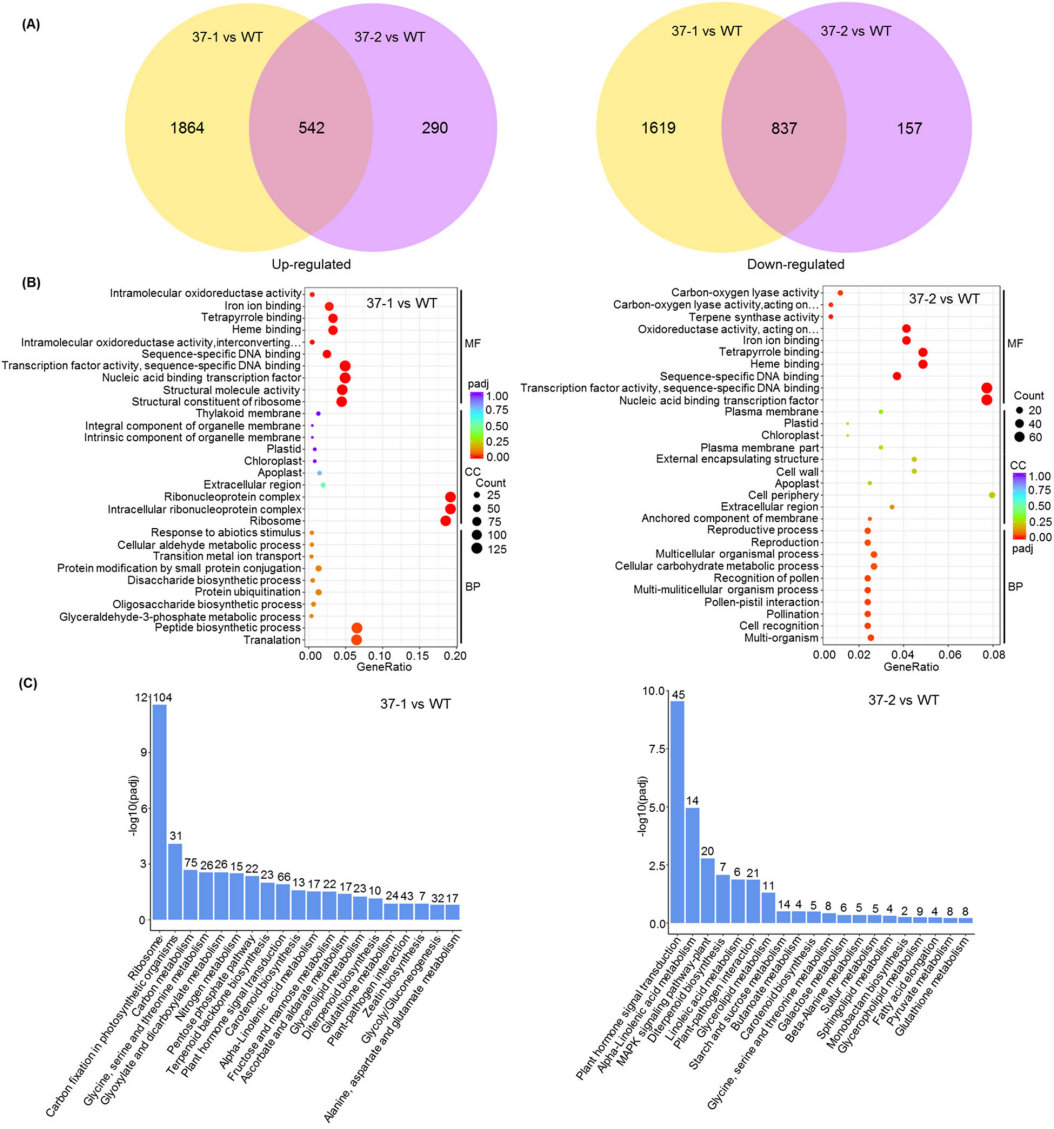
Overexpression of *MdVQ37* causes dramatic transcriptomic alterations in transgenic apple lines. **A** Venn diagram analysis of upregulated and downregulated DEGs in transgenic lines compared with WT plants. **B** Comparative GO enrichment analysis of the DEGs in the biological process (BP), cellular component (CC), and molecular function (MF) categories. **C** KEGG pathway analysis of the DEGs

The transcriptional differences between WT plants and *MdVQ37* transgenic lines were further characterized via transcriptome analysis (RNA-seq) of transgenic lines (37-1 and 37-2) and WT plants under normal conditions. After filtering, approximately 43.9 million clean reads were obtained for each genotype, with a total mapping percentage of 93.3% (Table S1). There were 1,379 differentially expressed genes (DEGs) (|log2(fold change)|≥ 1, padj ≤ 0.05), of which 837 were downregulated and 542 were upregulated in the 37-1 vs. WT and 37-2 vs. WT comparison groups, respectively (Fig. 6A; Table S2). To verify the accuracy of the RNA-seq data, 14 DEGs, including five upregulated and nine downregulated genes, were selected for transcript level measurements via qRT-PCR analysis (Fig. S4). The results of the qRT-PCR analysis of the expression of the 14 selected genes were consistent with transcript abundance as revealed by the RNA-seq data, suggesting that the DEG screening based on the RNA-seq analysis was reliable. Furthermore, we analyzed the expression patterns of these 14 DEGs under HT stress. Compared with WT plants, *MdVQ37* transgenic plants presented significantly lower transcript levels of *MdHSFA3*, *MdWRKY33*, *MdNAC2*, *MdMYB6*, *MdRAV1*, *MdPOD*, *MdMDHAR*, and *MdCAT* and increased transcript levels of *MdbHLH63* and *MdNAC87* under HT stress (Fig. S4). In particular, two genes involved in the ROS regulatory system, *MdCAT* and *MdMDHAR*, and genes involved in the AsA-GSH cycle were downregulated in transgenic plants, which was consistent with the measured enzyme activity of CAT and MDHAR under normal conditions and HT stress.

The identified DEGs in the 37-1 vs. WT and 37-2 vs. WT comparison groups were enriched in three major categories, namely, biological processes, molecular functions, and cellular components, which revealed transcriptional differences between the transgenic lines and WT plants. The enriched Gene Ontology (GO) terms in the biological process and cellular component categories were not similar in the 37-1 vs. WT and 37-2 vs. WT groups (Fig. 6B; Table S3). These groups shared seven enriched GO terms for the molecular function category: ‘transcription factor activity’, ‘nucleic acid-binding transcription factor activity’, ‘sequence-specific DNA binding’, ‘sequence-specific DNA binding’, ‘heme binding’, ‘tetrapyrrole binding’ and ‘iron ion binding’ (Fig. 6B). The top three GO terms, namely, ‘transcription factor activity’, ‘nucleic-acid binding transcription factor activity’ and ‘sequence-specific DNA binding’, were associated with DEGs that encoded various members of the TFs WRKYs, AP2/ERFs, MYBs, NACs, bZIPs, bHLHs, and HSFs (Table S3), which are known to regulate the stress response.

Kyoto Encyclopedia of Genes and Genomes (KEGG) pathway analysis of the DEGs from the 37-1 vs. WT and 37-2 vs. WT groups led to the identification of 20 major enriched pathways. The most highly enriched KEGG pathway was ‘plant hormone signal transduction’, followed by ‘alpha-linolenic acid metabolism’ (Fig. 6C; Table S4).

### *MdVQ37* overexpression leads to decreases in SA content and SA-dependent signaling pathways under heat stress

**Fig. 7:**
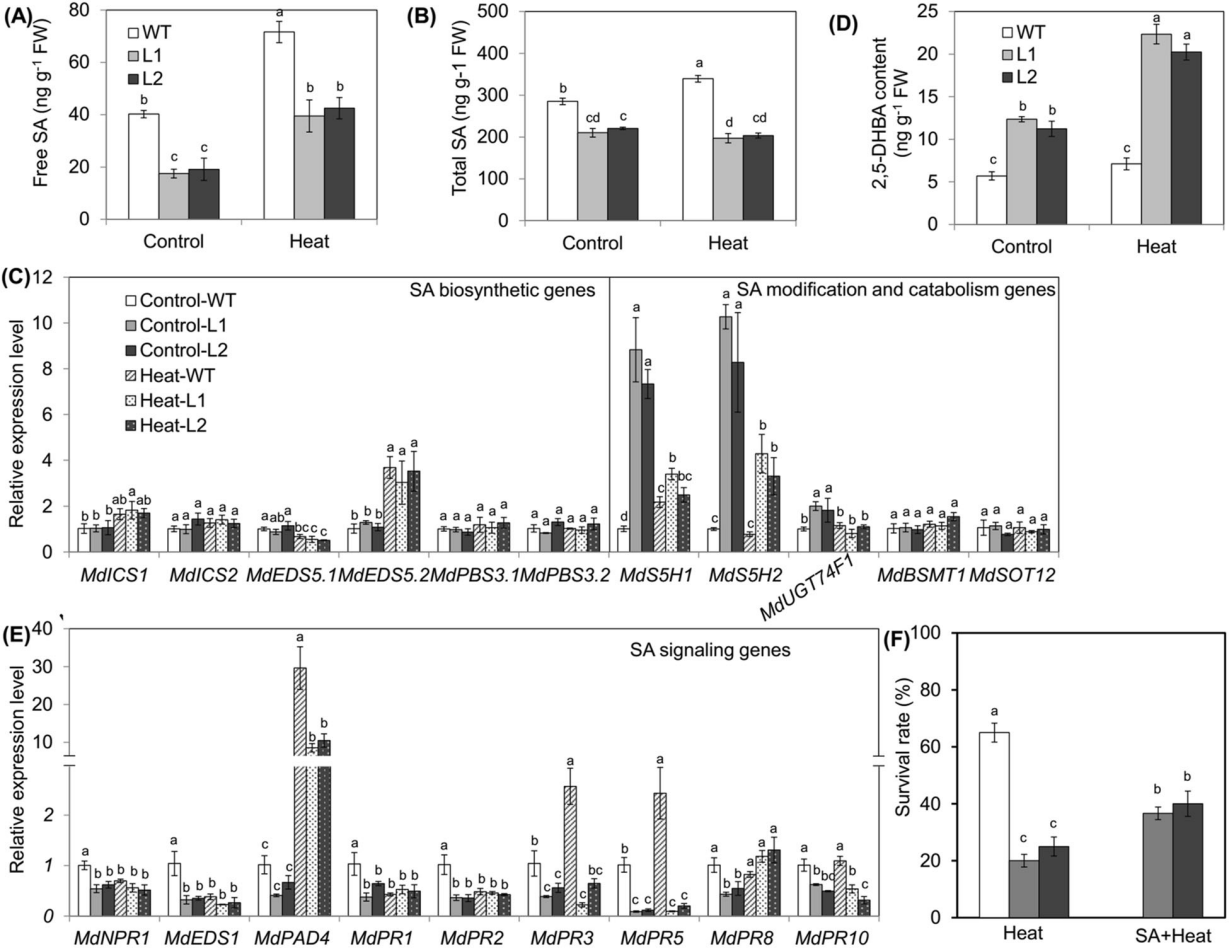
Effects of heat stress on endogenous SA levels, 2,5-DHBA contents, transcript levels of SA metabolism-related and signaling-related genes, and survival rates of WT plants and *MdVQ37* transgenic lines. The contents of endogenous free SA (**A**) and total SA (**B**) in WT plants and transgenic lines are subjected to HT stress or not. **C** Relative transcript levels of SA biosynthesis-related and catabolism-related genes under HT stress. **D** 2,5-DHBA content in WT plants and transgenic lines subjected to HT stress or not. **E** Relative transcript levels of SA signaling-related genes under HT stress. **F** The survival rate of transgenic lines sprayed with 100 μM SA under HT stress. The data represent the means ± SEs of three biological replications. The different letters represent significant differences among the treatments on each date (one-way ANOVA followed by Duncan’s test, *p* < 0.05)

As the analysis of the KEGG results revealed that the transgenic lines induced plant hormone signal transduction (Fig. 6C) and increased transcript levels of genes involved in SA signaling (Table S2), we measured the contents of endogenous SA in the WT plants and transgenic lines. The contents of endogenous free SA and total SA were higher in the WT plants than in the transgenic lines under normal-temperature conditions, suggesting that MdVQ37 affected endogenous SA homeostasis (Fig. 7A, B). After 4 h of HT stress, however, the free SA content drastically increased in both the WT plants and the transgenic lines, but the increase in the free SA content in the two transgenic lines was significantly lower than that in the WT plants, the levels of which were only 70.3 and 74.6% that of the WT plants (Fig. 7A). The total SA content exhibited a similar trend in the WT plants but not in the transgenic lines (Fig. 7B). To further understand why the endogenous SA content was reduced in the transgenic lines, we examined changes in the expression of SA biosynthesis-related and catabolism-related genes in the WT plants and transgenic lines. No difference in the transcript levels of SA biosynthesis genes was observed between plants of the different genotypes under normal-temperature conditions or HT stress (Fig. 7C). However, the expression of the SA modification-related and catabolism-related genes *MdS5H1* and *MdS5H2* in the transgenic lines was significantly higher than that in the WT plants (Fig. 7C). MdS5H1 and MdS5H2 encode salicylic acid 5-hydroxylase (S5H), also called 2-oxoglutarate-Fe(II) oxygenase, which catalyzes the conversion of SA into 2,5-dihydroxybenzoic acid (2,5-DHBA)^38^. Accordingly, we measured the content of 2,5-DHBA in the transgenic lines and WT plants. In contrast to the trend of free SA and total SA contents, the content of 2,5-DHBA in the transgenic lines was higher than that in the WT plants regardless of whether it was measured under normal-temperature conditions or HT stress (Fig. 7D). Furthermore, we measured the expression of SA signaling-related genes in the transgenic lines and WT plants. The expression of most of the SA signaling-related genes in the transgenic lines was lower than that in the WT plants under normal-temperature conditions (Fig. 7E). Among these genes, *MdPAD4*, *MdPR3,* and *MdPR5* were induced in response to heat stress, and the transcript levels of these three genes in the transgenic lines were significantly lower than those in the WT plants under HT stress (Fig. 7E), suggesting that *MdPAD4*, *MdPR3,* and *MdPR5* may play key roles in the resistance of apple to HT stress.

To determine whether suppression of the SA level in the transgenic lines was responsible for the impaired basic thermotolerance, the transgenic lines were pretreated with 100 µM SA. Although the survival rate increased, it was still lower than that of the WT plants (Fig. 7F). Therefore, the reduced basal thermotolerance of the transgenic plants stemmed in part from the decrease in SA levels.

## Discussion

Higher plants have different types of TFs that bind to cis-acting elements in the promoter regions of downstream genes and initiate their transcription^23,39^. VQ motif-containing proteins act as transcriptional regulators controlling a series of biological processes, such as plant growth, development, defense responses, and stress tolerance mechanisms. In model plant species, the biological functions of VQ motif-containing proteins in the response to abiotic stress have been less studied than their functions in the response to biotic stress have, especially HT stress. In fact, the link between VQ motif-containing proteins and basal thermotolerance has not yet been established in plants. Research on the functions of VQ motif-containing proteins in response to HT stress in plants aims to enhance our understanding of the various biological roles of VQ motif-containing proteins. In this study, we used transgenic apple plants overexpressing MdVQ37 to investigate the specific role of MdVQ37 in the response to HT stress. Our results clearly showed that MdVQ37 overexpression reduced tolerance to HT in transgenic apple lines (Fig. 1). HT stress resulted in more damage to *MdVQ37* transgenic apple lines than to WT plants through mechanisms involving increased EL, MDA, and ROS levels and decreased proline content, chlorophyll content, and photosynthesis activity (Figs. 1–5).

The accumulation of excessive amounts of ROS is a very important factor contributing to the many adverse effects of HT stress. Previous studies have shown that ROS-scavenging systems are essential for plants to cope with HT stress^4,9,40^. For instance, mutants lacking antioxidant pathways involved in ROS metabolism have been shown to be defective in basal thermotolerance^4^. In addition, overexpression of the apple ATG-encoding gene *MdATG18a* improved basal thermotolerance by enhancing antioxidant activity and reducing ROS accumulation^9^. In this study, we examined ROS regulatory systems in WT plants and *MdVQ37* transgenic lines and found that the activities of CAT, POD, SOD, APX, GR, DHAR, and MDHAR in *MdVQ37* transgenic lines were significantly lower than those in WT plants under HT stress (Figs. 2, 3). Consistent with the changes in the activities of ROS-scavenging enzymes, the total GSH and AsA levels, as well as the ratios of GSH/GSSG and AsA/DHA in the transgenic lines, were also significantly lower in the *MdVQ37* transgenic lines than in the WT plants under HT stress (Fig. 3). Therefore, both nonenzymatic and enzymatic ROS-scavenging regulatory systems were less active in the *MdVQ37* transgenic lines than in the WT plants under HT stress.

Several studies have also shown that a series of TFs, including HSFs, WRKYs, NACs, and bZIPs, are involved in the regulatory response of plants to HT stress. For example, *HSFA4A* overexpression enhances tolerance to HT stress by reducing oxidative damage^41^. *hsfA3* mutant lines were shown to have reduced thermotolerance during the HT stress response^13^. Heat stress-induced *WRKY39*, a subgroup IId WRKY member, positively regulates the cooperation between the JA-activated and SA-activated signaling pathways, both of which mediate responses to HT stress in *Arabidopsis*^12^. Functional analysis revealed that *wrky25* null mutants are sensitive to HT stress, while compared with WT *Arabidopsis* plants, transgenic plants overexpressing *WRKY25* were relatively tolerant to HT stress, suggesting that WRKY25 is a positive regulator of thermotolerance^42^. Guan et al.^14^ reported that the NAC TF NAC019 can regulate the expression of heat stress-responsive genes. Transgenic plants overexpressing *NAC019* have increased thermotolerance, and compared to WT plants, *nac019* mutants have reduced tolerance to HT stress. In addition, overexpression of the tomato NAC TF *SlJA2* reduced the content of endogenous SA, which led to significant changes in the physiology and gene expression patterns of transgenic tobacco plants, resulting in decreased heat tolerance of those plants^43^. In this study, the *MdHSFA4A* (MD09G1233700), *MdHSFA3* (MD14G1015900), *MdWRKY25* (MD11G1059400, MD12G1181000, and MD16G1066500), *MdWRKY39* (MD07G1146900), and *MdNAC019* (MD15G1136600 and MD15G1344900) genes, which are homologs of *Arabidopsis HSFA4A*, *HSFA3*, *WRKY25*, *WRKY39,* and *NAC019*, respectively, were significantly downregulated in the transgenic lines (Fig. 6, Table S2), suggesting that changes in the activities of many TFs in the transgenic lines negatively affected basal thermotolerance.

Previous studies have also shown that SA plays a key role in basal thermotolerance^44,45^. The SA-mediated signaling pathway has been shown to improve HT tolerance in *Arabidopsis*, cucumber, chickpea, grape, mustard, potato, tobacco, tomato, and wheat^12,18,42,46–49^. SA application can alleviate HT stress in grapes by inducing the activity of MDHAR, APX, and GR; increasing the redox ratio of AsA and GSH; and maintaining Ca^2+^ homeostasis under heat stress^48^. SA (1.0 mM) was shown to increase proline production and restrict stress-induced ethylene formation after heat stress^18^. Treatment with 0.5 mM SA was shown to decrease oxidative stress and EL and improve the maximum yield of PSII and Fv/Fm in cucumber seedlings under HT stress^47^. Thus, appropriate concentrations of SA can improve heat stress by affecting the ROS-scavenging system, the AsA-GSH cycle, Ca^2+^ homeostasis, and the photosynthetic system. In addition, multiple studies have demonstrated the importance of endogenous SA and SA signaling pathways in basal thermotolerance. For instance, SA-deficient *NahG* transgenic *Arabidopsis* plants, which have decreased endogenous SA contents, present reduced basal thermotolerance, whereas SA-accumulating *constitutive expressor of PR protein* (*cpr5*) mutants, which have increased endogenous SA contents, exhibit an enhanced basal thermotolerant phenotype^46,50^. The *Arabidopsis* mutants *npr1-1*, *npr1-5,* and *sid2*, which are defective in SA signaling, present decreased tolerance to heat stress^12,46,50,51^. Thus, endogenous SA content and the SA-dependent signaling pathway play important roles in basal thermotolerance. In this study, overexpression of *MdVQ37* led to significant changes in the transcriptional activity of multiple TFs, which caused a significant increase in the transcript abundances of the possible target genes *MdS5H1* and *MdS5H2*, resulting in significant accumulation of 2,5-DHBA in the transgenic lines (Figs. 6, 7). 2,5-DHBA is one of the most widely produced aromatic acids in green plants and is suggested to be a component of the major pathway for SA catabolism^38^. The overaccumulation of 2,5-DHBA disrupted the homeostasis of endogenous SA in the transgenic lines. SA accumulation is essential for activating downstream gene expression of SA and SA-dependent resistance^52^. Indeed, in this study, under HT stress, the expression of the SA signaling-related genes *MdPAD4*, *MdPR3,* and *MdPR5* in the *MdVQ37* transgenic lines were significantly lower than that in the WT plants (Fig. 7C, D). In this case, the transgenic lines exhibited reduced activities of ROS-scavenging enzymes (Figs. 2, 3), which attenuated AsA-GSH recycling (Fig. 3) and photosynthetic capacity (Figs. 4, 5) and ultimately led to reduced basal thermotolerance in the transgenic lines under HT stress. Moreover, spraying SA slightly increased the survival rate of the transgenic lines under HT stress (Fig. 7E), suggesting that the suppression of the SA level in the transgenic lines was partly responsible for the impaired basal thermotolerance. These findings also indicated that the impaired basal thermotolerance of the transgenic lines affected other signaling pathways.

**Fig. 8:**
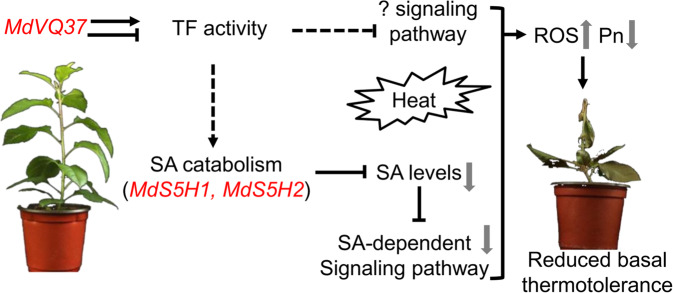
Putative working mode of the MdVQ37 regulatory function in the apple response to HT stress. As a transcriptional regulator, overexpression of *MdVQ37* can affect the transcriptional activity of multiple TFs, and indirectly up-regulated the expression of SA catabolism genes *MdS5H1* and *MdS5H2*. This causes reduction in endogenous SA content and disruption in SA-dependent signaling pathways. Moreover, increased expression of *MdVQ37* also resulted in unknown signal pathway. All of these changes resulted in higher ROS accumulation and lower photosynthetic capacity, and reduced basal thermotolerance under HT stress

In summary, we propose a putative working model of the regulatory function of MdVQ37 in the apple response to HT stress (Fig. 8). Overexpression of *MdVQ37*, a transcriptional regulator, can affect the transcriptional activity of multiple TFs and indirectly upregulate the expression of the SA catabolism-related genes *MdS5H1* and *MdS5H2*, causing a reduction in endogenous SA content and disruption of SA-dependent signaling pathways. Furthermore, the increased expression of *MdVQ37* triggered an as-yet-unknown signaling pathway. Together, all of these changes led to increased ROS accumulation, decreased photosynthetic capacity, and reduced basal thermotolerance under HT stress.

## Materials and methods

### Plant materials and heat treatment

GL-3 tissue culture-generated apple (*Malus domestica* ‘Royal Gala’) plants were used for genetic transformation to generate transgenic apple plants^53^. For expression analysis of *MdVQ* genes, 45-day-old healthy GL-3 apple plants were maintained in a growth chamber at 48 °C. After heat exposure for 1, 4, or 8 h, fully expanded apple leaves at the same position were collected for gene expression analysis of *MdVQ* genes. For heat stress exposure, healthy, 90-day-old transgenic lines and WT plants of uniform size were placed in a growth chamber at 48 °C for 2, 4, 6, or 24 h. In addition, transgenic lines and WT plants were maintained at room temperature (24 °C) as negative controls. After heat exposure for 2 and 4 h, the 8th and 9th leaves from the bottom of the stem of six plants were collected for analysis of the expression of genes involved in SA biosynthesis, modification and catabolism, and signal transduction and for the determination of the SA content. After heat exposure for 6 h, the 8th and 9th leaves from the bottom of the stem of six plants were harvested for damage assessment. All apple tissue materials were frozen immediately in liquid N_2_ and stored at −80 °C. For survival rate analysis, healthy, 90-day-old plants of uniform size (heat exposure) were maintained in a growth chamber at 48 °C for 24 h, followed by a 48-h recovery period at 24 °C. A plant was considered dead if 90% of its leaves failed to recover, and the plant survival ratio was determined (Fig. 1F). In addition, 90-day-old, healthy transgenic lines (SA + heat treatment) were sprayed with 100 μM SA solution, and control plants were sprayed with water. At 24 h after pretreatment, the plants were maintained in a growth chamber at 48 °C for 24 h, followed by a 48-h recovery period at 24 °C, and the plant survival ratio was determined (Fig. 7F).

### Construction of plasmids and genetic transformation of apple

We used RT-PCR to isolate the coding region of *MdVQ37* from mature leaves of Royal Gala apple plants to construct an *MdVQ37* overexpression vector^31^. The coding region was inserted into a pCAMBIA2300 plant transformation vector driven by the cauliflower mosaic virus (CaMV) 35S promoter. *Agrobacterium*-mediated genetic transformation of apple leaves was performed with GL-3 as the genetic background and EHA105 as the infectious strain, as described previously^53^. Regenerated buds were screened with 25 mg/L kanamycin monosulfate (Kan). Kan-resistant seedlings were subcultured every month in subculture media. The transgenic lines were then confirmed via genomic PCR amplification analysis (Table S5). The overexpression of *MdVQ37* was evaluated via qRT-PCR analysis (Table S6).

### Evaluation of stress tolerance

The EL of the apple leaves was analyzed as previously described^54,55^. The levels of proline and MDA were measured using PRO-2-Y and MDA-2-Y kits (Comin, Suzhou, China), respectively, according to the manufacturer’s instructions. The chlorophyll concentrations were determined according to a previously described method^56^.

### Antioxidant enzyme activities and histochemical staining

The levels of oxidized glutathione (GSSG), GSH, AsA, DHA, H_2_O_2_, and O_2_.− and the activities of CAT, POD, SOD, GR, DHAR, APX, and MDHAR were analyzed using the following test kits purchased from Comin: GSSG-2-W, GSH-2-W, AsA-2A-W, DHA-2-W, H_2_O_2_-2-Y, SA-2-G, CAT-2-Y, POD-2-Y, SOD-2-Y, APX-2-W, GR-2-W, MDHAR-2-W, and DHAR-2-W, respectively. The manufacturer’s instructions were followed for each kit. After 6 h of heat exposure, fresh solutions of NBT and DAB were used to stain the leaf samples with O_2_.− and H_2_O_2_, respectively. The stained leaves were immersed in a 70% ethanol decolorizing solution at 95 °C in a water bath for 4 h and then transferred to a 10% glycerin solution for observations and imaging.

### Evaluation of photosynthetic characteristics and chlorophyll fluorescence

After a 24-h recovery period following 6 h of heat exposure, leaf Pn, Tr, Ci, and g_s_ were measured using a CIRAS-3 portable photosynthesis system (PP System, Amesbury, MA, USA)^9^. Transient chlorophyll fluorescence was examined using an Open FluorCam FC 800-O instrument, and Fv/Fm ratios were calculated using Fluorcam7 software (PSI, Brno, Czech Republic)^9,57^. For all heat exposure experiments, photosynthetic characteristics and chlorophyll fluorescence were measured on the 6th and 7th leaves from the bottom of the stems of each selected plant.

### Measurement of stomatal aperture

After 4 h of heat exposure, fully expanded leaves were removed from the same position of each selected plant and immediately cut into small square pieces (0.5 × 0.5 cm). The samples were subsequently immersed in a 4% glutaraldehyde solution to avoid any alteration or damage during sample preparation and maintained at 4 °C for 24 h. The stomata were then observed via scanning electron microscopy (SEM) with a JSM-6360LV scanning electron microscope (JEOL, Ltd., Tokyo, Japan), as previously described^56^.

### Measurements of energy conversion and electron transport in PSI and PSII

After a 24-h recovery period, following 6 h of heat exposure, the PSI [Y(I) and ETR(I)], and PSII [Y(II) and ETR(II)] activities of the apple leaves were measured simultaneously using a Dual-PAM-100 system (Heinz Walz GmbH, Effeltrich, Germany) in conjunction with a Fluo C P700 instrument as previously described^9,57^.

### RNA preparation, transcriptome sequencing, and differentially expressed gene analysis

For transcriptome analysis, 90-day-old leaves (eighth leaf) from the bottom of the stem of healthy plants were collected for RNA extraction. Total RNA from three groups of leaves (WT, 37-1, and 37-2) was isolated using a DP441-RNA Prep Pure Plant Plus Kit (Tiangen, Beijing, China) according to the manufacturer’s instructions. Illumina RNA sequencing was then performed by Novogene Bioinformatics Technology Co., Ltd. (Beijing, China) on an Illumina HiSeq 4000 platform, following previously described methods^58^. DEGs with a fold change ≥2 and an FDR ≤ 0.05 were identified using the DESeq2 R package (1.16.1)^59^. KEGG pathway enrichment analysis and GO enrichment analysis of the DEGs were performed using cluster Profiler (3.4.4) software^60^.

### RNA isolation and qRT-PCR analysis

Total RNA from apple leaves was isolated using a Wolact Plant RNA Isolation Kit (Wolact, Hong Kong, China). cDNA was synthesized using a PrimeScript First-Strand cDNA Synthesis Kit (TaKaRa, Dalian, China). qRT-PCR was performed on a LightCycler® 96 quantitative instrument (Roche Diagnostics, Basel, Switzerland) in conjunction with TransStart Top Green qRT-PCR SuperMix (Transgen Biotech, Beijing, China), as described previously^31^. Three biological replications were used for each assay, and the *MdMDH* gene was used as an endogenous control to normalize the transcript levels of different genes (Table S6)^61^. The gene expression was determined using the 2^*−*ΔΔCt^ method^62^.

### Metabolite extraction

SA was extracted and purified according to previously described methods^63,64^. Briefly, after accurately weighing 0.1 g of apple leaves, the leaves were ground in liquid nitrogen, suspended in 1 mL of methanol, and incubated at −20 °C overnight. Subsequently, the suspension was centrifuged at 13,000 rpm for 15 min at 4 °C, the supernatant was air-dried with nitrogen gas, and the extract was dissolved in 200 μL of 80% methanol. Then, a 100-μL aliquot of the extract was air-dried under a stream of nitrogen gas and resuspended in 500 μL of sodium acetate buffer (0.1 M; pH 5.5). A 200 μL aliquot of the suspension was used to analyze the free SA content via liquid chromatography-mass spectrometry (LC-MS). In addition, a 200-μL aliquot of the suspension was treated with 10 μL of β-glucosidase, incubated in a water bath at 37 °C for 2 h, and then boiled in water for 5 min to stop the enzymatic reaction. Afterward, the suspension was centrifuged at 13,000×*g* for 10 min at 4 °C, after which the supernatant was subjected to LC-MS analysis to determine the total SA content. 2,5-DHBA was extracted according to previously described methods^65^. Briefly, after accurately weighing 0.1 g of apple leaves, the leaves were ground in liquid nitrogen, suspended in 1 mL of extraction solution (methanol:water:formic acid (25:24:1)), subjected to ultrasonic treatment for 20 min (normal temperature, 40 Hz, 100 W), centrifuged at 12,000 rpm for 15 min at 4 °C, and then filtered through a 0.25 μm filter membrane. The filtrate was ultimately subjected to LC-MS analysis to determine the 2,5-DHBA concentration.

### Liquid chromatography-mass spectrometry (LC-MS) analysis

The LC-MS technique was used as described previously^64^. A QTRAP^®^ 5500 LC-MS/MS system (Applied Biosystems/MDS Analytical Technologies, Foster City, California, USA) equipped with an InertSustain AQ-C18 column (4.6 × 150 mm, 5 mm; GL Sciences, Torrance, CA, USA) was used to analyze the metabolites. The mobile phase consisted of 0.1% (v/v) formic acid (A) and methanol (B). To measure the SA content, a gradient elution process was implemented through the following steps: 75% A (0 min), 75% A (1 min), 5% A (5 min), 5% A (6.5 min), 75% A (6.6 min), and 75% A (13 min). For the measurement of the 2,5-DHBA content, the gradient elution process was implemented as follows: 75% A (0 min), 65% A (1 min), 5% A (6 min), 5% A (8.9 min), and 75% A (9 min). The flow rate of the aliquot was maintained at 0.7 mL min^−1^ throughout the process. The concentrations of free SA, total SA, and 2,5-DHBA were analyzed by calculating the LC-MS peak area based on standard curves.

### Statistical analysis

All the data were obtained from three biological replications and analyzed with IBM SPSS (version 20) statistical software (IBM Corporation, Armonk, NY, USA). The experimental data are expressed as the mean values ± standard errors (SEs). One-way analysis of variance (ANOVA) was performed to compare significant differences between the WT plants and transgenic lines based on Duncan’s test (*p* < 0.05).

## Supplementary information

Expression analysis of *MdVQ*s under HT treatment

PCR identification and relative expression analysis of *MdVQ37* in *MdVQ37* overexpressing transgenic lines and WT plants

Phenotypes of *MdVQ37* transgenic lines and WT plants after 24 h HT treatment and recovery for 48 h

Summary of RNA-sequencing data for the three replicates of each genotype

Primers used for vector construction and detection

Primers used for qRT-PCR

Differential gene expression analyses of fourteen selected DEGs under normal condition and heat stress

Go analysis in transgenic lines

KEGG pathways analysis in transgenic lines

DEGs in transgenic lines
