# Supplementary material for: MdVQ37 overexpression reduces basal thermotolerance in transgenic apple by affecting transcription factor activity and salicylic acid homeostasis
Source: Hortic Res. 2021 Oct 1;8:220. doi: 10.1038/s41438-021-00655-3 (PMC8484266; doi:10.1038/s41438-021-00655-3)
Supplement: Supplementary file 1 — Expression analysis of MdVQs under HT treatment [file 41438_2021_655_MOESM1_ESM.docx]

Figure S1. Expression analysis of *MdVQ*s under heat treatment. *MdHSFA2*, as control gene, was used to respond to heat stress. 45-day-old, healthy GL-3 apple plants of uniform size were kept at 48 °C in a growth chamber. Data are shown as the means of three biological replicates with SEs. Different letters indicate significant differences between treatments, according to one-way ANOVA and Duncan’s tests (*p* < 0.05)..
