# Supplementary material for: MdVQ37 overexpression reduces basal thermotolerance in transgenic apple by affecting transcription factor activity and salicylic acid homeostasis
Source: Hortic Res. 2021 Oct 1;8:220. doi: 10.1038/s41438-021-00655-3 (PMC8484266; doi:10.1038/s41438-021-00655-3)
Supplement: Supplementary file 2 — PCR identification and relative expression analysis of MdVQ37 in MdVQ37 overexpressing transgenic lines and WT plants [file 41438_2021_655_MOESM2_ESM.docx]

Figure S2. PCR identiﬁcation and relative expression analysis of *MdVQ37* in *MdVQ37* overexpressing transgenic lines and WT plants. M, DNA marker; -, negative control (H_2_O); +, positive control (plasmid DNA of 35S:MdVQ37 pCambia2300 vector). Specific primer for *MdVQ37* was used to detect relative expression levels of *MdVQ37* overexpressing transgenic lines and WT plants. For details regarding specific primers, refer to Table S5 and S6.
