# Supplementary material for: MdVQ37 overexpression reduces basal thermotolerance in transgenic apple by affecting transcription factor activity and salicylic acid homeostasis
Source: Hortic Res. 2021 Oct 1;8:220. doi: 10.1038/s41438-021-00655-3 (PMC8484266; doi:10.1038/s41438-021-00655-3)
Supplement: Supplementary file 3 — Phenotypes of MdVQ37 transgenic lines and WT plants after 24 h HT treatment and recovery for 48 h [file 41438_2021_655_MOESM3_ESM.docx]

Figure S3. Phenotypes of *MdVQ37* transgenic lines and WT plants after 24 h heat treatment and recovery for 48 h.
