# Supplementary material for: MdVQ37 overexpression reduces basal thermotolerance in transgenic apple by affecting transcription factor activity and salicylic acid homeostasis
Source: Hortic Res. 2021 Oct 1;8:220. doi: 10.1038/s41438-021-00655-3 (PMC8484266; doi:10.1038/s41438-021-00655-3)
Supplement: Supplementary file 4 — Summary of RNA-sequencing data for the three replicates of each genotype [file 41438_2021_655_MOESM4_ESM.docx]

**Table S1.** Summary of sequencing data for each sample.

| sample | raw_reads | clean_reads | total_map | unique_map | multi_map | read1_map | read2_map | positive_map | negative_map | splice_map | unsplice_map | proper_map |
| --- | --- | --- | --- | --- | --- | --- | --- | --- | --- | --- | --- | --- |
| WT-1 | 44653260 | 43604132 | 40834715(93.65%) | 39819614(91.32%) | 1015101(2.33%) | 19937277(45.72%) | 19882337(45.6%) | 19907118(45.65%) | 19912496(45.67%) | 15474648(35.49%) | 24344966(55.83%) | 38353762(87.96%) |
| WT-2 | 44515216 | 43595298 | 40707118(93.38%) | 39667365(90.99%) | 1039753(2.39%) | 19864447(45.57%) | 19802918(45.42%) | 19831919(45.49%) | 19835446(45.5%) | 15383830(35.29%) | 24283535(55.7%) | 38134080(87.47%) |
| WT-3 | 43161408 | 42104376 | 39320961(93.39%) | 38348030(91.08%) | 972931(2.31%) | 19190749(45.58%) | 19157281(45.5%) | 19169449(45.53%) | 19178581(45.55%) | 14927635(35.45%) | 23420395(55.62%) | 36889200(87.61%) |
| 371-1 | 44672254 | 43694646 | 40811103(93.4%) | 39828084(91.15%) | 983019(2.25%) | 19950869(45.66%) | 19877215(45.49%) | 19905482(45.56%) | 19922602(45.6%) | 15738019(36.02%) | 24090065(55.13%) | 38260944(87.56%) |
| 371-2 | 45956982 | 44956590 | 41988002(93.4%) | 40933340(91.05%) | 1054662(2.35%) | 20480881(45.56%) | 20452459(45.49%) | 20455264(45.5%) | 20478076(45.55%) | 16298074(36.25%) | 24635266(54.8%) | 39350758(87.53%) |
| 371-3 | 42937826 | 41906754 | 39002845(93.07%) | 38002773(90.68%) | 1000072(2.39%) | 19036998(45.43%) | 18965775(45.26%) | 18991697(45.32%) | 19011076(45.37%) | 14762556(35.23%) | 23240217(55.46%) | 36564266(87.25%) |
| 372-1 | 45307930 | 44267152 | 41347069(93.4%) | 40370658(91.2%) | 976411(2.21%) | 20210711(45.66%) | 20159947(45.54%) | 20174481(45.57%) | 20196177(45.62%) | 15366191(34.71%) | 25004467(56.49%) | 38857552(87.78%) |
| 372-2 | 49875362 | 48580646 | 45006713(92.64%) | 43888843(90.34%) | 1117870(2.3%) | 21976440(45.24%) | 21912403(45.11%) | 21942808(45.17%) | 21946035(45.17%) | 16795774(34.57%) | 27093069(55.77%) | 42212902(86.89%) |
| 372-3 | 43924224 | 43099292 | 40301874(93.51%) | 39319777(91.23%) | 982097(2.28%) | 19680738(45.66%) | 19639039(45.57%) | 19651335(45.6%) | 19668442(45.64%) | 15423678(35.79%) | 23896099(55.44%) | 37957628(88.07%) |

Note: “371” means MdVQ37 overexpressing apple line 1

“372” means MdVQ37 overexpressing apple line 2,

“WT” means wild type apple.
