# Supplementary material for: MdVQ37 overexpression reduces basal thermotolerance in transgenic apple by affecting transcription factor activity and salicylic acid homeostasis
Source: Hortic Res. 2021 Oct 1;8:220. doi: 10.1038/s41438-021-00655-3 (PMC8484266; doi:10.1038/s41438-021-00655-3)
Supplement: Supplementary file 5 — Primers used for vector construction and detection [file 41438_2021_655_MOESM5_ESM.docx]

Table S5. Primers used for vector construction and detection

| Primer name | Primer sequences |
| --- | --- |
| 2300-MdVQ37F | GAGAACACGGGGGACTCTAGAATGGAAAGACAT |
| 2300-MdVQ37R | GGGAAATTCGAGCTCGGTACCTTAGGAGGACGG |
| 2300F | GAGAACACGGGGGACTCTAGA |
| 2300R1 | CGATCGGGGAAATTCGAGCTC |
| 2300R2 | TGCCAAATGTTTGAACGATC |
