# Supplementary material for: MdVQ37 overexpression reduces basal thermotolerance in transgenic apple by affecting transcription factor activity and salicylic acid homeostasis
Source: Hortic Res. 2021 Oct 1;8:220. doi: 10.1038/s41438-021-00655-3 (PMC8484266; doi:10.1038/s41438-021-00655-3)
Supplement: Supplementary file 6 — Primers used for qRT-PCR [file 41438_2021_655_MOESM6_ESM.docx]

Table S6. Primers used for qRT-PCR.

| Name | Gene ID | Forward primer sequences | Reverse primer sequences |
| --- | --- | --- | --- |
| *MdVQ1* | MD15G1373500 | CACCACCACCACCAACACTCAC | GGAAGAGGAAGCGGAGGAGGAG |
| *MdVQ2* | MD08G1187500 | CTCCTCCTCCACTTCCTCTTC | GCGGAAGTCATTTTTGTTGATG |
| *MdVQ3* | MD15G1338100 | TGTCCTTAGTCCAACGCCTCACC | CGGCCACGACCTTGTTGTAATCC |
| *MdVQ4* | MD01G1011800 | AAATGGGTCAATTTCCGGGC | TGGCCATCCCCATCCAATTA |
| *MdVQ5* | MD05G1018300 | CAACTCCAAGGTCCACGTCCAAC | TGGTTGTTGTGACGGTTGTGGTAC |
| *MdVQ6* | MD10G1018000 | TGGTGGTGGTGGTGGTGATGG | GAAGGAATCGGAGTAAGCGAAGCG |
| *MdVQ12* | MD06G1101200 | CCACCTCCAGCTCCTCTTCCTG | GCGGCATTGGTGTAGGTGATGG |
| *MdVQ13* | MD14G1120900 | GCGCAAACCTACTTACAGAAAA | TTTCTCCGTGTATTTGGTACGA |
| *MdVQ14* | MD08G1181100 | ACCGAAGCACGAGGAGGACAG | GTGGTGAGAACGGTGGTTGGTG |
| *MdVQ15* | MD15G1366700 | TGGCAGCTTCAGATGGCAATGATG | AGTCCACCGCACCTTCACCTC |
| *MdVQ16* | MD01G1011800 | CTCGCCTCCGTACACAAGACAAG | CCTCCTCCTCCGCCTCCTTG |
| *MdVQ19* | MD10G1293000 | AACAGCAACCACCACCTCAACG | TGAAGGAGGAGGTGTCAGCTTGG |
| *MdVQ20* | MD16G1041600 | CCTTCAAACAAGTCGTCCAAAT | GAGACGGGGATTAATGGGTTAA |
| *MdVQ25* | MD14G1058100 | ATGGAGGAGGCTCAGACGCTAAC | TCCAGTGAGTCTCTGAACCAGCTC |
| *MdVQ26* | MD04G1191900 | AAGCAGCAGAGAAGCGTGATTCC | GTGACCTGTCCGTTACCGAATCTG |
| *MdVQ31* | MD08G1052300 | TCAAGCTGCAAGAGCGAAGACAC | AGACGACGGCGACTCGGAAC |
| *MdVQ34* | MD17G1215500 | TGAAGAGGAAGATCGAGCCATTGC | GCTCAGGAGTGTGGTCAGTCATTC |
| *MdVQ37* | MD15G1043200 | GGTGGCGGAGGAGGACAAGG | AGGAGGACGGCAGAGACTTGTG |
| *MdVQ38* | MD03G1229000 | TCTGGGTTTTCTCCACGTCA | AATCCTTTCTCCGCAATCGC |
| *MdVQ39* | MD01G1027700 | AACTCAACTACTCGGCTGCT | CGCTTCGAGGAGTGGTAGTA |
| *MdVQ42* | MD11G1249800 | GTCCAAGCTGACTCCTCGAACTTC | GTGGTGGTGGTGGCTGTTGTG |
| *MdVQ43* | MD15G1325900 | CTTCATTGCTTGATCCCGTTAG | CTGAGTCAAGCCAAAATGTTGA |
| *MdVQ44* | MD03G1229000 | TCTGGGTTTTCTCCACGTCA | AATCCTTTCTCCGCAATCGC |
| *MdHSFA2* | MD15G1057700 | GAATGCCAAGAATAGAGCACTG | TAGTCCACAACGTCGATTCTAG |
| *MdMDH* | MD16G1219000 | CGTGATTGGGTACTTGGAAC | TGGCAAGTGACTGGGAATGA |
| *MdMDHAR* | MD09G1030800 | CCTGTTTGAAGGTCAACTAACG | TTCCATAATTGCAGTAACAGCG |
| *MdHSFA3* | MD14G1015900 | ATTGACGGGTGGATTGTTAGAT | GGCTCATCAAATGCAGATTCAT |
| *MdCAT* | MD06G1008700 | AAACCGAACCCTAAGTCCCA | AGCCTTCCATGTGCCTGTAA |
| *MdbZIP50* | MD06G1002100 | AAGAGTGGGTCAAAGGCCAA | ATTAGAGGAGGGAGGGCAGA |
| *MdPOD* | MD03G1014400 | GGTTGTGATGGATCGATTATGC | AATGTCAGAACAGGAGACAACA |
| *MdMYB6* | MD13G1026400 | CGACCGAACAACGTTAATCTTT | CTTGCTGCTGATAATTGCTAGG |
| *MdWRKY33* | MD12G1181000 | GTACGAAGGGAAGCACAACC | GTTGGCTTTCAGTGGTTGCT |
| *MdNAC2* | MD15G1136600 | GGGACAGAAAATATCCAAACGG | CTTTTTAATCCCGAGTGCCTTG |
| *MdRAV1* | MD16G1047700 | AAAGTGTGGAGGTTTCGATACT | TATACAGATGTTTGTCCGGTCC |
| *MdbHLH63* | MD10G1074000 | AACCGAGCTTTACCCGAGAT | GAAAACCCCACTAAGCTCGC |
| *MdCAX5* | MD04G1151600 | TATGATAGGAGACAAGCTGACG | CACAGCTGGAAAACTATGTACG |
| *MdARF4* | MD11G1132400 | TTTCAACGTTTATCAACCACCC | GTAGCATTAGAGCACATTGTGG |
| *MdNAC87* | MD12G1001200 | TACAACTTTCAGCTGCATCCTA | ATTGCTCATCATCTGATTTGGC |
| *MdSBP9* | MD12G1060000 | TGCTGCTTTGGACCTTGATG | CCCAAGGTGTTATTCAAATCGC |
| *MdICS1* | MD06G1188700 | TTTCCGCCCCATAGTCCTTG | ACAACCCCAGTTCCAGCATAG |
| *MdICS2* | MD14G1195500 | GCGCTCTTTCGTGGACTTTG | TATTGCACTCCACTCCAGCA |
| *MdEDS5.1* | MD05G1056900 | GTAAAGCGTAGTTTGCCTAAGG | TGGTCGGGTGTAAATATACCAG |
| *MdEDS5.2* | MD10G1065100 | CAAACTGCACAATCGTTTATGC | ACATGTTCCGACAATCCCTAAT |
| *MdPBS3.1* | MD13G1132300 | AGAACGGTTGATCTAGTGGATG | TCTGTACCTGTATAGCCCTGTA |
| *MdPBS3.2* | MD16G1142800 | GGAATCAATTTCAACCCGCTAA | TCCATTAGATCAACCGTTTCCA |
| *MdS3H1* | MD09G1188300 | CTACGGTATGGAGAAGTACCAC | TCGAACATTATCCACTCGTCAT |
| *MdS3H2* | MD03G1070400 | GATAAGCCTCTCCACAGCTATT | GTTTCTGGATTCTCAATGCCAG |
| *MdBSMT1* | MD10G1071200 | CAATGCCTGGTTCTTTCTATGG | GTGACCTCAAAAACACCGTAAA |
| *MdSOT12* | MD16G1056100 | TGTTTGGAGTTCGATGTCTACA | CAACCCGAATGTTTGACAGAAT |
| *MdUGT74F1* | MD02G1003300 | CTGTTTGGAAGGTTGGTGTTAG | TTTCCTAATGCAGCGCTTAATC |
| *MdNPR1* | MD10G1236700 | ATTTTGTTGGGAAAGCTCTGTC | TCATCAATATCTGATCGTGCCA |
| *MdEDS1* | MD14G1188700 | CGAGCCGCAATACCCATATC | TCATCTGGGAATTTGTGCGC |
| *MdPAD4* | MD15G1136300 | GACAGCCATGCAACCAGTTT | AATTTGAAGCTGGACGTGGC |
| *MdPR1* | MD05G1109100 | GCAGCAGTAGGCGTTGGTCCCT | CCAGTGCTCATGGCAAGGTTTT |
| *MdPR2* | MD12G1002300 | CCCAAGTTCCCAGTCGATCT | ACCATATTCCAATGCCGTGC |
| *MdPR5* | MD09G1256000 | AACTAGCATCCAAAGCTAGCC | CCACAGTCTGCAGTTTCACAAG |
| *MdPR3* | MD07G1282800 | CAAAGGACACAACGGACAGG | GCATTGGGCTAGGGGGTTG |
| *MdPR8* | MD01G1213300 | GCCACCGAAACTTACTCTCTCAC | CAACTCAATGTCGAAATCAACGC |
| *MdPR10* | MD13G1160700 | CACCTCCGTCATCCCCCCTGC | CCTTGTCAACCCCATCAATTCTGTG |
