# Supplementary material for: MdVQ37 overexpression reduces basal thermotolerance in transgenic apple by affecting transcription factor activity and salicylic acid homeostasis
Source: Hortic Res. 2021 Oct 1;8:220. doi: 10.1038/s41438-021-00655-3 (PMC8484266; doi:10.1038/s41438-021-00655-3)
Supplement: Supplementary file 7 — Differential gene expression analyses of fourteen selected DEGs under normal condition and heat stress [file 41438_2021_655_MOESM7_ESM.docx]

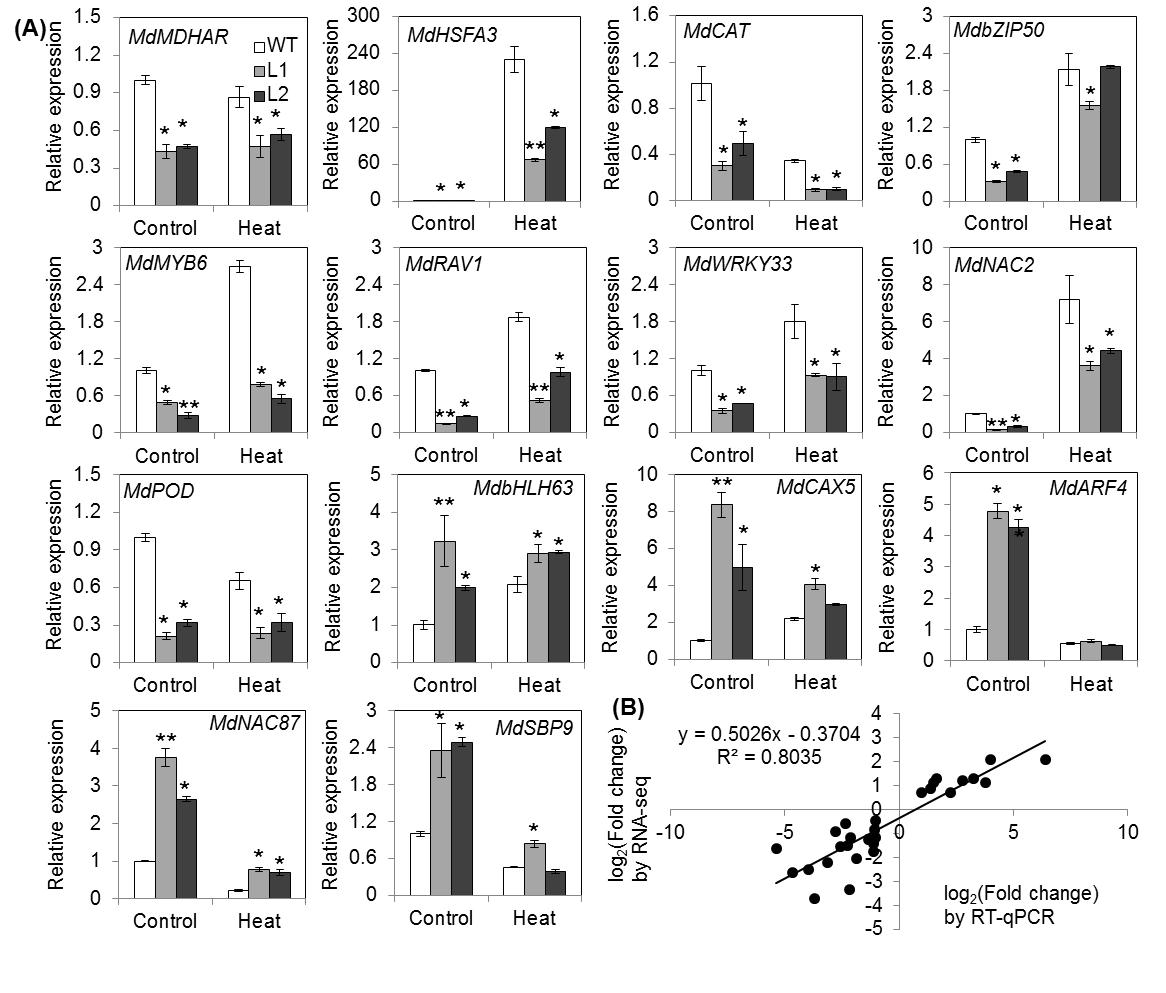


Figure S4. Differential gene expression analyses of fourteen selected DEGs under normal condition and heat stress. (A) Transcript levels of fourteen selected DEGs, as revealed by RT-qPCR. 3-month-old, healthy plants of uniform size were kept at 48 °C for 2 h in a growth chamber, and plants at a normal temperature (24 °C) were used as the negative control. After 2h of heat treatment, the eighth leaves from the base of the stems of six plants were collected for expression analyses. Error bars indicate standard deviation (SD) for three biological replicates. One-way ANOVA (Duncan’s test) was performed and statistically significant differences were indicated by *(*P* < 0.05) or **(*P* < 0.01). For details regarding specific primers, refer to Table S1. (B) Correlation of expression analysis by RNA-seq (Y-axis) and RT-qPCR (X-axis).
